# AI-enabled photonic smart garment for movement analysis

**DOI:** 10.1038/s41598-022-08048-9

**Published:** 2022-03-08

**Authors:** Leticia Avellar, Carlos Stefano Filho, Gabriel Delgado, Anselmo Frizera, Eduardo Rocon, Arnaldo Leal-Junior

**Affiliations:** 1 Graduate Program in Electrical Engineering, Federal University of Espírito Santo (UFES), Fernando Ferrari Avenue, Vitória, 29075-910 Brazil; 2 Neurophysics Group, “Gleb Wataghin” Institute of Physics, University of Campinas, Campinas, Brazil; 3 Centro de Automática y Robótica, Ctra. Campo Real, 28500 Arganda del Rey, Madrid Spain

**Keywords:** Biomedical engineering, Optical sensors, Electrical and electronic engineering

## Abstract

Smart textiles are novel solutions for remote healthcare monitoring which involve non-invasive sensors-integrated clothing. Polymer optical fiber (POF) sensors have attractive features for smart textile technology, and combined with Artificial Intelligence (AI) algorithms increase the potential of intelligent decision-making. This paper presents the development of a fully portable photonic smart garment with 30 multiplexed POF sensors combined with AI algorithms to evaluate the system ability on the activity classification of multiple subjects. Six daily activities are evaluated: standing, sitting, squatting, up-and-down arms, walking and running. A k-nearest neighbors classifier is employed and results from 10 trials of all volunteers presented an accuracy of 94.00 (0.14)%. To achieve an optimal amount of sensors, the principal component analysis is used for one volunteer and results showed an accuracy of 98.14 (0.31)% using 10 sensors, 1.82% lower than using 30 sensors. Cadence and breathing rate were estimated and compared to the data from an inertial measurement unit located on the garment back and the highest error was 2.22%. Shoulder flexion/extension was also evaluated. The proposed approach presented feasibility for activity recognition and movement-related parameters extraction, leading to a system fully optimized, including the number of sensors and wireless communication, for Healthcare 4.0.

## Introduction

In recent years, technologies such as Internet of Things (IoT) have been employed as strategical approaches for decentralized decision making through the connection of the digital and physical worlds^1^. The IoT concept stems from the interconnection of “things” in which the data communication between heterogeneous devices is performed without any human assistance^2^. The main requirements of IoT include high data rate, real-time (low-latency) operation, high security, long battery lifetime, connection density and mobility^3,4^. Smart Healthcare is an IoT application which aims at the improvement of the everyday quality of life in the end-user community^5^. Sensor devices are employed to collect medical data and vital signs from patients to monitor health conditions, track progress and indicate anomalies^2^. Moreover, remote healthcare monitoring with high speed and intelligent execution can be achieved by increasing the number of devices and using Artificial Intelligence (AI) algorithms^6^, since the combination of IoT and AI in the healthcare sector has a higher potential of making intelligent decisions in real-time for patient medical records^5,7^.

Motor activities and physiological condition are important information for individual’s health monitoring. Gait-related parameters are useful in fall detection, gait pattern characterization or balance assessment^8^. Changes in the gait-related parameters can indicate gait abnormalities or even a motor impairment, which can be further investigated. The motor impairment caused by injuries or diseases can be regarded as a limitation of muscle control function or mobility limitation^9^. Bilateral limb analysis can be useful in the evaluation of the changes in motor ability, such as in the case of motor impairment after stroke, in which the control of limbs movement of one side of the body is affected. Among the gait-related parameters, cadence monitoring (i.e. number of steps per minute) can provide valuable information regarding user’s gait and activities in a remote monitoring environment, where deviations in the cadence can be related to fatigue of the patient or, in some cases, it can be related physical and even cognitive impairment^10^. Although the cadence can be estimated with different approaches, such as accelerometers, camera-based systems and even laser range finders, the use of an integrated and fully portable solution such as the one proposed in this work provides benefits for remote healthcare applications, or any applications outside a clinical environment, due to the possibility of monitoring the patients in different activities of their daily living using portable systems that do not restrict their natural pattern of their movements^11^. Besides the biomechanical parameters, the acquisition of physiological parameters is indispensable in the physiological analysis and provides important information about movement performance. Breathing rate is a physiological parameter that consists of the number of breathing cycles (inhalation and exhalation) per minute. Continuous monitoring of the breathing rate can help to detect pulmonary diseases at their early stage or help to evaluate the level of physical conditioning and movement fatigue^11^. Breathing rate monitoring can be an indicator of different clinical conditions such as infections (can result in the increase of carbon oxide concentration, leading to faster breathing rate^12^), narcotics and even alcohol abuse^12^. For this reason, many technologies were developed for breathing rate monitoring. Among the wearable ones, there are smart textiles and chest belts based on electronic and optical technologies, which provide the breathing rate estimation from vibrations or chest displacements^13^. However, such sensors not only include another clothing or wearable accessory in addition to the one the patient is already using, but also have deviations on the measurement depending on the sensors’ position and are sensitive to movements of the users^13^. Such drawbacks are mitigated using a smart garment, where there is the integration of all sensors in a single clothing accessory, which can also detect the breathing rate at different regions under dynamic movements of the user.

Flexible sensors miniaturization and advancements have boosted the development of wearable technologies to track health-related parameters or to extract practical features from multi-modal sensors on the wearable device^6,14^. Wearable sensors play an important role in remote healthcare monitoring, since they allow performing a diagnostic evaluation at home with the use of non-invasive and unobtrusive sensors during daily activities^11^. There are popular wearable devices in the market, such as inertial sensors embedded in elastic bands, smart watches and instrumented insoles, for movement and posture analysis, physiological parameters monitoring and pressure plantar detection^14^. However, simultaneous monitoring of different health-related parameters requires the use of several individual devices, which may lead to problems related to devices’ connection and synchronization, in addition to a discomfort and a possible skin irritation due to long-term use of these wearable devices^15^. Sensors integration with clothing, so-called smart textile, is an attractive solution to overcome these drawbacks. The smart textiles present the advantages of sensors compactness and higher transparency between the sensor and the user, which leads to the monitoring of the natural activity without inhibiting the user’s movement^16^. Furthermore, smart textiles are easily handled, with simple installation and removal, which represents an advantage in terms of usability. Several approaches have been proposed for the development of smart textile technologies, including conventional electronics^17^, photonics^18^ and optical fiber sensing^19^.

Optical fiber sensors (OFS) have attractive features for smart textile technology, including compactness, lightweight and multiplexing capabilities. In addition, OFS are not susceptible to electrical discharges and they are immune to electromagnetic interference^15^. The polymer optical fiber (POF) sensors have additional advantages since they present high flexibility and good biocompatibility with the human body^20^. Different sensing techniques are used in the development of POF sensors, such as Fiber Bragg gratings (FBGs)^21^ and interferometry^22^, which are commonly used in the scientific literature due to their high sensitivity to strain and temperature. Such technologies are also embedded in textiles for medical parameters monitoring, where sensors for body temperature, breathing rate, oximetry, heart rate and movement monitoring were proposed^20^. However, these wavelength-based techniques generally involve non-portable and expensive optical units for the signal acquisition^20,23,24^. It is also important to mention that portable technologies for FBGs interrogator generally have limitations on the number of channels or simultaneous sensors^25^. An alternative approach is the transmission-reflection analysis (TRA) embedded in textiles which presents portability and low-cost components^26^. Nevertheless, this approach is also not fully optimized for remote healthcare monitoring since it includes a large number of components and it does not enable simultaneous multi-point analysis.

Most of these drawbacks can be addressed using the intensity variation technique, which consists of a light source and a photodetector to measure optical transmission losses due to the strain in the POF, resulting in a simpler and low-cost technique with compact components^27,28^. Such technique is also used in the development of optical fiber-embedded textiles^20^. However, the intensity variation setup using one light source and one photodetector (one at each fiber end) results in one sensor per fiber^29^. The employment of many sensors requires a high number of fibers, photodetectors, sources and acquisition electronics units, reducing the system compactness and limiting the flexibility of the sensor system, which is unfeasible for wearable applications in the remote health monitoring. A multiplexing technique was proposed to solve this issue by coupling light-emitting diodes (LEDs) to different fiber lateral sections and using temporal modulation to achieve multiplexed sensors positioned in different locations in the same fiber^30^. Thus, the proposed photonic smart textile combines the advantages of multiplexing capabilities for quasi-distributed sensor systems development of FBGs with the low cost and portability of intensity variation-based sensors, which enable the development of a low-cost, textile-integrated and fully portable sensor array for simultaneous assessment of multiple parameters.

The fundamental purpose of AI is to create intelligent machines which simulate the human intelligence and learn to better mimic the human process to revolutionize the area of decision-making^7,31^. Machine Learning is a branch of AI that learn from existing ”training” data to make predictions about new data points^7^. Most applications of machine learning in healthcare involve supervised machine learning methods, which consist of algorithms that are trained on ”ground truth” labels, e.g. support vector machines (SVM), random forests, k-nearest neighbors (kNN)^32^. Deep Learning is a subclass of Machine Learning that uses artificial neural network architectures, containing a large number of layers that resemble the structure of human cognitive functions and connect and direct data^33^. With more complex models, decision-making can be more accurate when Deep Learning algorithms are employed. However, Deep Learning is better suited to analysing disordered and complicated data, whereas Machine Learning algorithms, such as kNN, are popular methods with simple implementation, significant classification performance and real-time implementability for more robust data^34^.

Combining the low-cost wearable approach based on multiplexed POF-based sensors with AI algorithms, this paper presents a promising remote healthcare monitoring solution based on a scalable photonic garment capable of accurately identifying activities, by using the kNN classification algorithm, and assess movement-related parameters, including physiological and spatio-temporal gait parameters, for application in Smart Healthcare. The photonic smart garment consists of the sensors composite structures, which are made of the light source and optical fiber encapsulated with a clear urethane mixture, integrated to a textile and distributed between the trunk regions. The proposed approach has compact acquisition electronics with simple processing and the possibility of including heterogeneous devices in the system. Moreover, the proposed system includes wireless communication which leads to a high potential of making intelligent real-time decisions in a homecare assessment, eventually. This paper also presents an optimization technique of the number of sensors incorporated in the garment using the Principal Component Analysis (PCA) technique based on the sensors’ weight during the activities performed in the kNN classification process.

## Results

**Figure 1:**
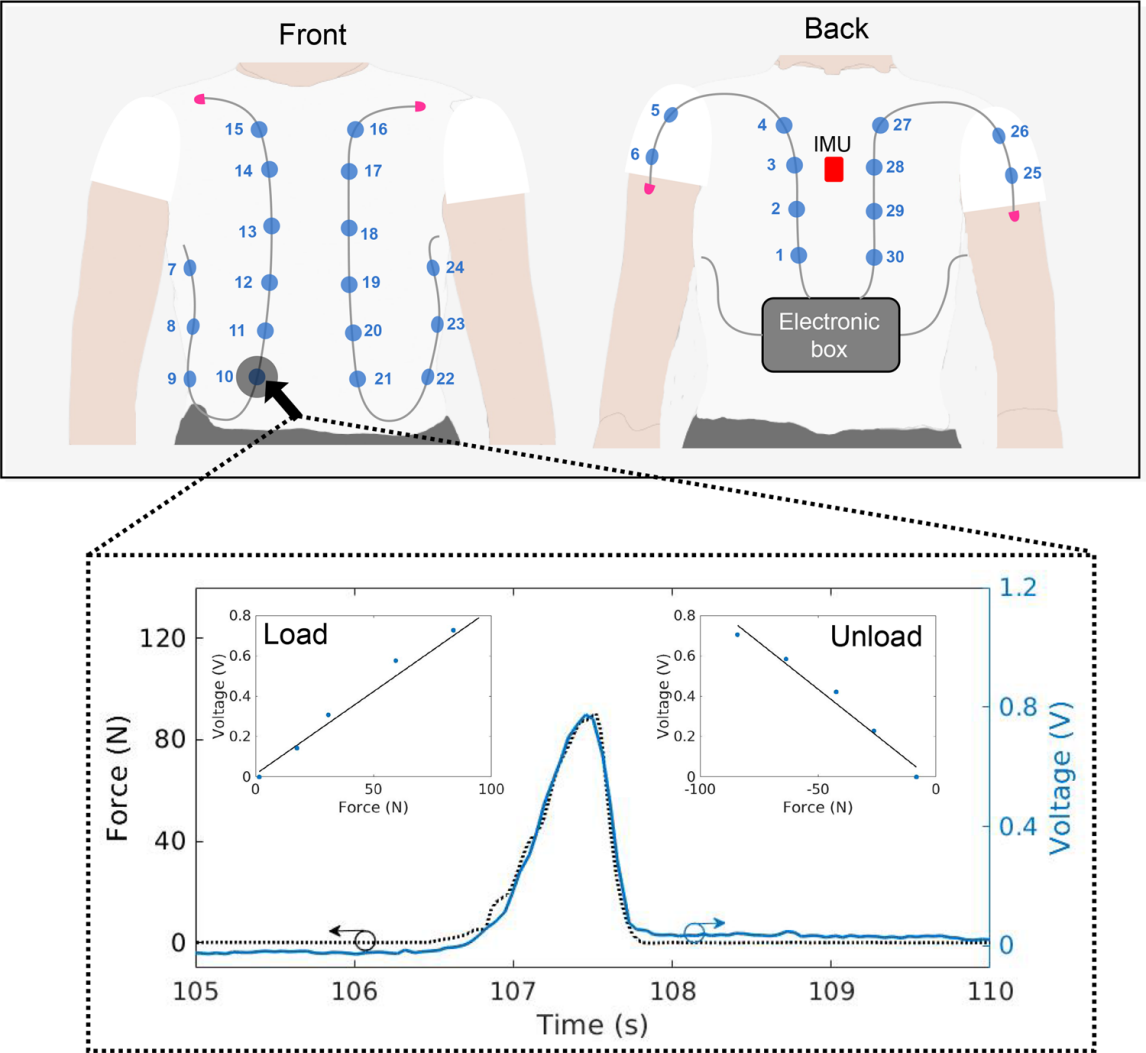
Photonic Smart Garment overview and the sensor response when a force is applied on the top of the sensor 10.

The proposed system comprises the photonic smart garment using sensors composite structures based on OFS technology. The photonic smart garment consists of 30 intensity variation-based POF sensors distributed in 4 fibers and integrated in a textile. In order to obtain an uniform distribution of the sensors in the smart garment, adjacent sensors are positioned with 10-cm distance between them to evaluate the possibility of identifying and classify activities using this sensor arrays. The sensors are numbered as shown in Fig. 1 in sequential order from the back to the front of the smart garment. In order to identify the number of sensors required for activity detection, we implemented a PCA to verify the sensors with the highest impact on the activity classification, i.e., the sensors with highest optical signal variation considering all performed activities. This process leads to a sensor system with a structure optimized as a function of minimum number of sensors needed to correctly classify the movements. In this case, the stress or strain applied on the optical fiber leads to a variation in the transmitted optical power. This phenomenon enables to infer the stress or strain in the OFS through the optical power variation. In addition, as discussed in Methods section, there is a side coupling between the light emitting diode (LED) and the optical fiber with a lateral section (exposing its core to the LED) using a clear urethane rubber in-between. Thus, a transverse loading (stress or strain) also results in a variation of the distance between the LED and optical fiber, which also leads to optical power variation. For the sensors multiplexing, the technique proposed in previous works^30^ was used, where there is a sequential activation of each LED in which only one LED is activated at a time during 1 *ms*. The optical power detection is synchronized with the LED activation. Each LED is attached to the sensitive zone of the fiber (lateral section). Therefore, each LED is related to one sensor and the optical signal acquisition when each LED is active results in an acquisition matrix in which each column represents the one sensors (i.e., there are 30 columns in this case) and each row represents a sample. The 30 sensors arranged in the garment are presented in Fig. 1. The sensors are characterized by applying different forces on each one and all sensors presented similar behavior. Figure 1 (figure inset) illustrates the response of one sensor (sensor 10) while forces are loaded and unloaded. The sensor response during the loading and unloading presents a determination coefficient ($R^2$) of 0.9941 and 0.9865, which indicates high linearity. Furthermore, the loading and unloading curves of the sensors are analyzed to estimate the sensors hysteresis, calculated from the deviations between the loading and unloading curves. In this analysis, the estimated hysteresis of the sensors is around 1.16% with a standard deviations between sensors of 0.81%. Based on the sensor noise power and the signal power, the signal-to-noise ratio (SNR) is estimated as 22*dB*. The data communication is performed by Bluetooth and all the system electronics is powered by a battery, resulting in a fully portable system. The acquisition frequency of the system is 100 Hz, which reduce the issues of unstable and discontinuous data acquisition. At this sample rate the classification algorithm is able of classifying the activity even when there is a minor reduction of the samples. In addition, the system has a Secure Digital (SD) card for the data storage for offline analysis. As a result, the system is also capable of offline analysis even if there are losses of packages and/or connectivity in the Bluetooth device used in the real-time monitoring.

**Figure 2:**
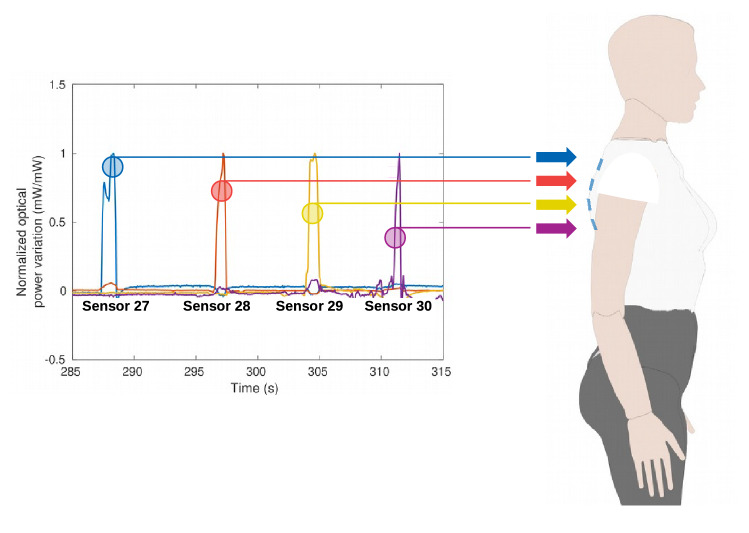
Response of sensors 27-30 when a predefined loading is applied to each sensor.

The sensors presented physical differences resulting from the manufacturing process (as mentioned in the Methods section), and as a result, they do not present the same sensitivity. To address this issue, the sensors’ responses are normalized by their sensitivities, which leads to similar responses on each sensor. The multiplexing capability of the sensors is confirmed by sequentially applying the same force on adjacent sensors (27, 28, 29 and 30). Figure 2 shows a significant difference between the response of the sensor in which the force was applied, and the responses of the adjacent sensors that indicate a negligible cross-talk considering the low optical power variations of the other sensors. The classification is then performed through machine learning algorithm, where the inputs of the algorithm is the normalized optical power variation of each sensor, since the multiplexing technique leads to negligible cross-talk between sensors.

**Figure 3:**
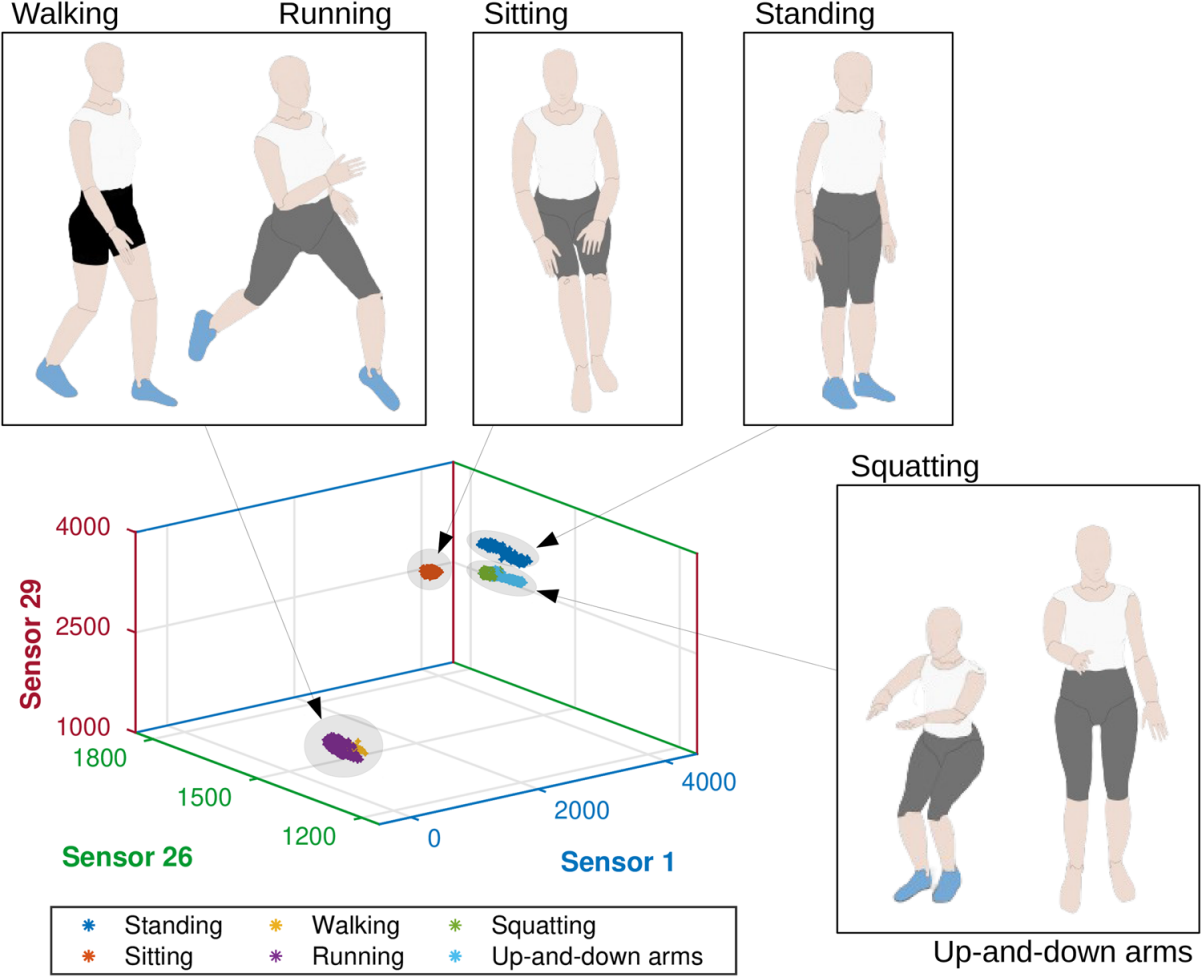
Clustering of six classes (activities) using the response of 3 sensors.

Figure 3 shows the responses of 3 arbitrary sensors (1, 26 and 29) positioned on different places of the body divided into 6 classes (activities). Differences between some classes, such as the $c_1$ (standing) and $c_2$ (sitting), are highly perceptible, which can be verified from the variations on the sensors responses (in ADC units) presented at each axis of the graph. Each point of the curve represents the responses of sensors 1, 26 and 29 for each activities in different tests. The clustering of each movement shown in Fig. 3 represents the repeatability and signal robustness of our approach, where there are similar responses for each activity when performed in different tests. As the sensors responses are proportional to the strain in the fiber, the variations on the sensors responses at each movement (or activity condition) depends on which regions of the user’s body are under strain for the performed movements This could be used in clinical scenarios for biomechanical analysis to quantify rehabilitation, surgical intervention and movement-related pathology^5^. Some of the classes are completely separated from the other ones by using only these 3 sensors’ responses. Standing is a stationary activity, with no movement, which does not involve pressure on any sensor. Sitting is also a stationary activity, however it involves the hip flexion and the activation of the back sensors. Since Fig. 3 presents the sensors’ responses of two sensors located on the back (1 and 29), the variation of these sensors leads to a separation of the data related to the $c_2$ (sitting).

The classes $c_3$ (squatting) and $c_4$ (up-and-down arms), as well as $c_5$ (walking) and $c_6$ (running), present similar clusters when the responses of these 3 sensors are evaluated. The activities of the classes $c_3$ (squatting) and $c_4$ (up-and-down arms) are not similar. Class $c_3$ (squatting) represents the squatting activity, which involves a knee flexion/extension and a hip flexion/extension. On the other hand, class $c_4$ (up-and-down arms) represents the up-and-down arms, which involves a great shoulder flexion/extension and a short longitudinal rotation. However, since there are no significant variation in the trunk movement, the responses of the two selected sensors (1 and 29) located on the back are similar for both movements. On the other hand, the response of the sensor 26 (located on the left arm) presents a bigger movement range for $c_4$ (up-and-down arms). The activities that represent classes $c_5$ (walking) and $c_6$ (running) comprise gait-related movements, yielding similar movement pattern of the limbs (hip, trunk and arms) which influence the smart garment sensors. The significant differences consist of the speed and the intensity level of the movement. The analysis of the sensors responses in conjunction with their positioning in the body lead to the possibility of estimating the relationship between the human body and sensor responses. Considering the sensors presented in Fig. 3 (i.e. sensors 1, 26 and 29), for activity/class 1 ($c_1$, standing), all analyzed sensors presented their highest optical power (considering all performed activities), which is related to the fact that the sensors are not under bending (or other mechanical loading). This is because the bending leads to attenuation in the transmitted optical fiber. For activity $c_2$ (sitting), there is an attenuation on the optical signals of sensors 1 and 29, which are the sensors positioned on the back of the user (see Fig. 1), which is related to the back support when the volunteer sits on a chair. The sensor 26 (in the arms region) does not presented major variations in activity $c_2$. In activity $c_3$ (squatting), the sensor 26 presents optical power attenuation due to the arms movement during this activity represented in Fig. 3. There are minor optical power variations in Sensors 1 and 29 (positioned on the user’s back) related to spine movements during squat activity. Similarly, up-and-down arms movements (activity $c_4$) also results in only minor variations in sensors 1 and 29, whereas the sensor 26 (in the user’s arm) presented higher optical power variation, since the bending and displacement occurs directly in the arms region. Walking ($c_5$) and running ($c_6$) activities resulted in the highest variations of all sensors, which is related to the movements in the upper and lower limbs in such activities, where there are spine movements that lead to bending in sensors 1 and 29 as well as the arms movement during gait, resulting in optical power variations in sensor 26.

This 3-dimensional evaluation is limited by the analysis of 3 sensors’ responses to illustrate a graphical result, which may lead to a weak classification performance if only these 3 sensors are used, since visually some classes contain overlapping data. However, in the classification process, the responses of the 30 sensors are initially employed until the minimal number of sensors is identified. The kNN dataset was randomly permuted and divided into training (70%) and testing (30%). Although it is possible to use other training/testing dataset divisions, such as 80/20 or 90/10, the proposed training/testing division is preferred for the size of the employed dataset, since the limit of 70% of the training dataset mitigates overfitting issues resulting in high accuracy for the testing data. The training data were the base data to classify the testing data, i.e., each new sample was compared with all training data. Thus, the predominant label of the k nearest samples was defined as the label of this new sample, and this was applied to all testing data. The euclidean distance was used as distance metric. The kNN algorithm was chosen mainly due to its lower computational cost (when compared with other classification algorithms) that enable its use in real-time monitoring in embedded systems. On top of that, the kNN presents high accuracy on the movement classification, which leads to a balance between the accuracy and computational cost suitable for fully-integrated systems.

The model evaluation metrics were divided into accuracy, recall and precision. Furthermore, the confusion matrix of each trial was analyzed. Accuracy refers to the percentage of samples correctly classified. Recall represents the percentage of samples of a class correctly classified with respect to all samples of that class, whereas precision represents the percentage of samples of a class correctly classified with respect to all samples predicted for that class. The results of the classifications for each volunteer are presented in Table 1. The accuracies of 10 random trials for all the volunteers dataset are higher than $90\%$. The different classification accuracies may be related to the movements performed by each volunteer during the activities of the protocol, since each person perform the movements in a particular way. This means that volunteer 1 performs movements more expressive for each activity which facilitates the classification, whereas volunteer 3 presents more error in activities classification, mainly involving the walking and running activities which are the movements most similar to each other of this protocol. In addition, the recall and precision results are presented in Table 1. It is possible to observe that some classes present more success in the classification than others. For all volunteers, the classes $c_1$ (standing) and $c_2$ (sitting) present a great difference when compared with the other classes. This also confirms the previous analysis in which only three sensors (1, 26 and 29) were analyzed. Differently, the data regarding classes $c_3$ (squatting) and $c_4$ (up-and-down arms), and the data regarding $c_5$ (walking) and $c_6$ (running) are different for each volunteer, which is related to the individual movement of each person.

**Table 1:** Classification results for each volunteer.

Volunteer	1	2	3	4
Accuracy (%)	99.96 (0.04)	92.04 (0.45)	91.34 (0.36)	94.86 (0.25)
**Recall (%)**				
$c_1$	100 (0.00)	98.31 (0.36)	99.50 (0.18)	99.91 (0.03)
$c_2$	100 (0.00)	99.56 (0.19)	100 (0.00)	100 (0.00)
$c_3$	100 (0.00)	89.69 (0.77)	94.25 (0.62)	89.77 (1.17)
$c_4$	100 (0.00)	89.93 (1.40)	99.98 (0.04)	92.98 (0.75)
$c_5$	99.87 (0.00)	93.94 (0.69)	82.62 (1.98)	94.50 (0.66)
$c_6$	99.87 (0.00)	78.69 (1.73)	64.91 (1.61)	91.46 (1.22)
**Precision (%)**				
$c_1$	100 (0.00)	96.02 (0.71)	94.92 (0.60)	98.34 (0.46)
$c_2$	100 (0.00)	96.56 (0.66)	100 (0.00)	100 (0.00)
$c_3$	100 (0.00)	92.08 (1.01)	98.97 (0.20)	91.56 (1.04)
$c_4$	100 (0.00)	91.67 (0.63)	100 (0.00)	93.48 (0.84)
$c_5$	99.87 (0.00)	82.50 (1.21)	69.48 (1.15)	91.77 (1.03)
$c_6$	99.87 (0.00)	92.44 (0.72)	79.53 (1.91)	93.19 (0.44)

By analyzing the volunteer 1, who presented the best classification results, it is possible to notice that the walking ($c_5$) and running ($c_6$) classes presented higher classification errors, which resulted in a recall and precision of 99.87 (0.00)%, for both classes. Gait speeds for walking and running activities are different, and hence the intensity level of the movement changes, resulting in a different variation of the sensors’ responses. However, the gait cycles for walking and running activities are similar, thereby the sensors’ responses present a similar pattern and, for this reason, some samples are misclassified. Similarly, the squatting ($c_3$) and up-and-down arms ($c_4$) activities are related to similar upper limbs movements and presented misclassified samples.

**Figure 4:**
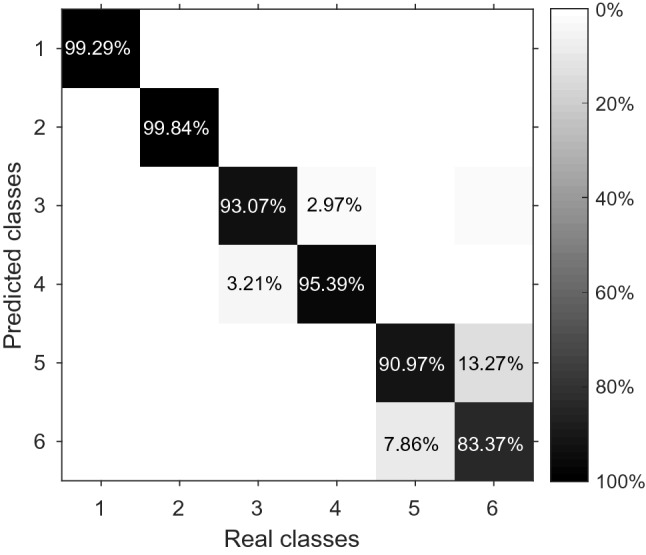
Confusion matrix regarding 10 trials including the dataset of all volunteers in the kNN classification.

Data from each volunteer are combined into a single dataset to analyze overall system classification performance, yielding an average accuracy (across participants) of 94.00 (0.14)%. Moreover, by analyzing the confusion matrix, it is possible to observe the influence and classification success of each class. Figure 4 shows the confusion matrix of the kNN classification for the whole dataset, including all volunteers. On the diagonal of each matrix are the percentage of the samples correctly classified. The data that resulted in the confusion matrix of Fig. 4 are presented as Supplementary Information, Supplementary Table S1.

**Figure 5:**
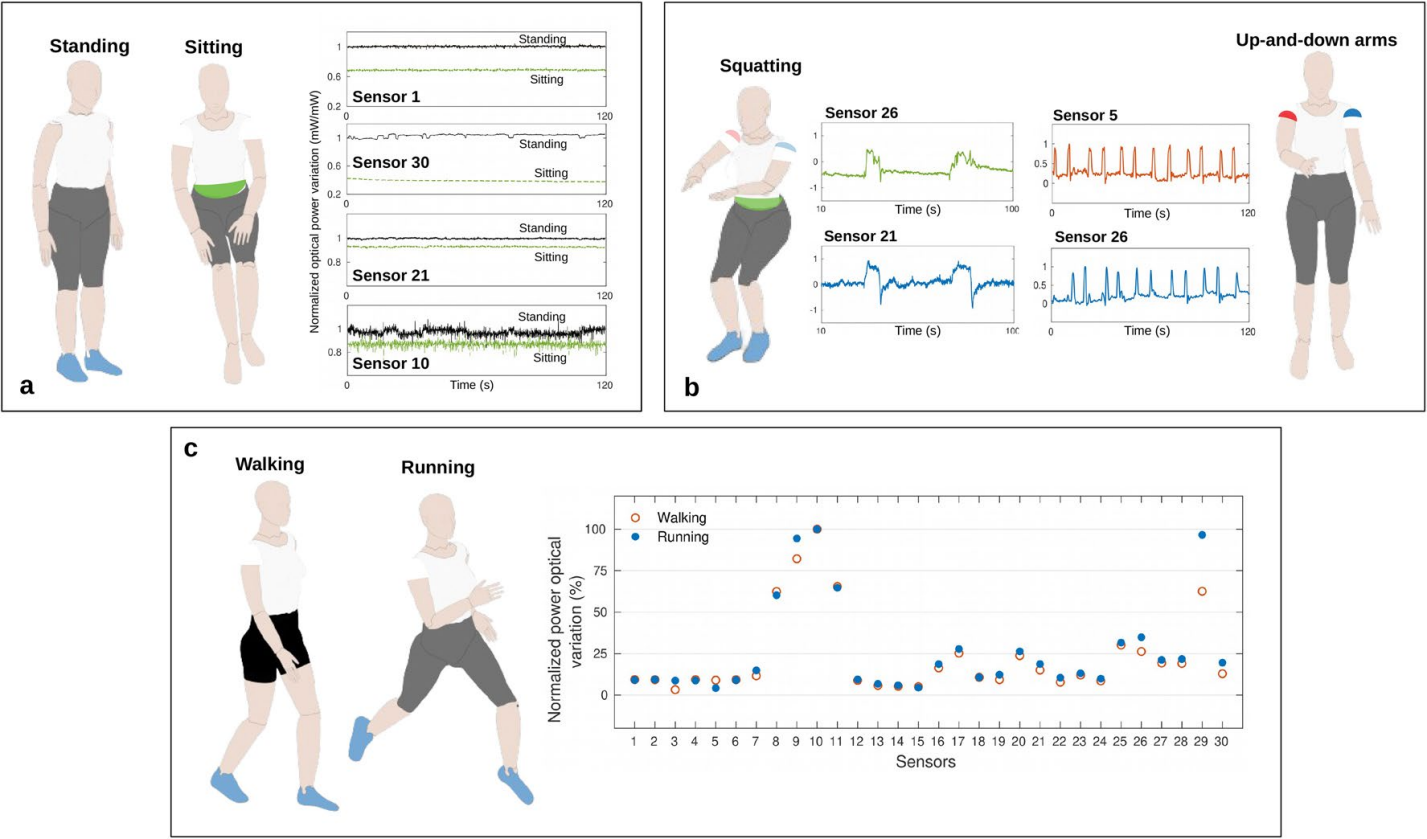
Sensors activation for each activity. (**a**) Optical power variation between the standing and sitting activities of sensors 1, 30, 21 and 10. (**b**) Response of sensors 26 and 21 during the squatting activity and response of sensors 5 and 26 during the up-and-down arms activity. (**c**) Sensors activation during the walking and running activities.

Depending on the activity, some sensors are not activated, i.e., they do not present a significant optical power variation when compared to the sensors’ response in the standing activity. The activation of different sensors for each activity occurs due to the fact that the activities consist of the movement combination of different body regions. Figure 5 presents the sensors activation for each activity. The results in Fig. 5 illustrate the variations of the transmitted optical powers of the sensors with the highest optical signal variation among the 30 sensors in the garment. In this case, the sensors are normalized to compare the optical power variation as a function of time for the presented sensors. In the sitting activity there are variations in the response of sensors located on the back and lower region of the trunk (sensors 21 and 10), close to the user’s hip, since this activity involves the hip flexion of approximately 90$^\circ$ and the contact of the user’s back to a chair (sensors 1 and 30), as shown in Fig. 5a. Figure 5b shows the temporal response of the most activated sensors during the squatting (sensors 26 and 21) and up-and-down arms (sensors 5 and 26). In sitting and squatting activities the sensor positioned on the hip region (sensor 21) is activated and this is due to the high hip flexion during both activities. In the same way, the sensor positioned close to the shoulder (sensor 26) is activated during the squatting, since the volunteers used the arms to achieve the balance of the body. During the up-and-down arms activity the sensors positioned on the right and left shoulders (sensors 5 and 26) presented the highest normalized optical power variation and this is due to the flexion and extension of each arm which leads to a high bending in the fiber. The walking and running activities comprise the intercalated legs movement, which involves the cyclic hip rotation, and hence, a higher optical variation in the response of sensors located on the lateral and close to the hip. Figure 5c presents the percentage of the optical power variation of each sensor during walking and running.

As Fig. 5 shows the sensors with the highest optical power variations, the sitting activity presents a high optical power variation on the sensors positioned on the back of the user, especially sensors 1 and 30 which are the ones closest to the lumbar region of the user’s. This region is exposed to the highest force/stress (when compared with the other sensors in the back region). It is important to mention that in the standing activity none of the sensors are under bending or stress. The standing activity is used as a comparison with the sitting activity, where there is a significant variation in the transmitted optical powers. In contrast, the squatting and up-and-down arms activities present major differences between them, such differences are translated in the positions of the sensors with the highest optical power variation for these activities. In the squatting activity, sensors 21 and 26 presented the highest optical power variations (among the ones in the garment). It is important to notice that sensors 21 and 26 are positioned in the user’s abdomen and arm, respectively, as shown in Fig. 1). Such regions have higher activation since the mechanics of squatting movement include trunk and arm movements, leading to the activation of the sensors in these regions. Moreover, the up-and-down arms movements lead to higher optical power variations in the sensors positioned in the arms (close to the shoulder, as presented in Fig. 1). Thus, the highest optical power variation was obtained in sensors 5 and 26, placed on the shoulders of the user. Both sensors followed the periodic movement of the up-and-down arms activity. Finally, for running and walking activities, some sensors presented high optical signal variation, as shown in Fig. 5c, where the highest variations were found in sensors 8-11 and 29. These sensors are positioned in the abdomen (sensors 8-11) and lower back regions (sensor 29), which track the variations related movements in the trunk during the walking and especially the running activity.

**Figure 6:**
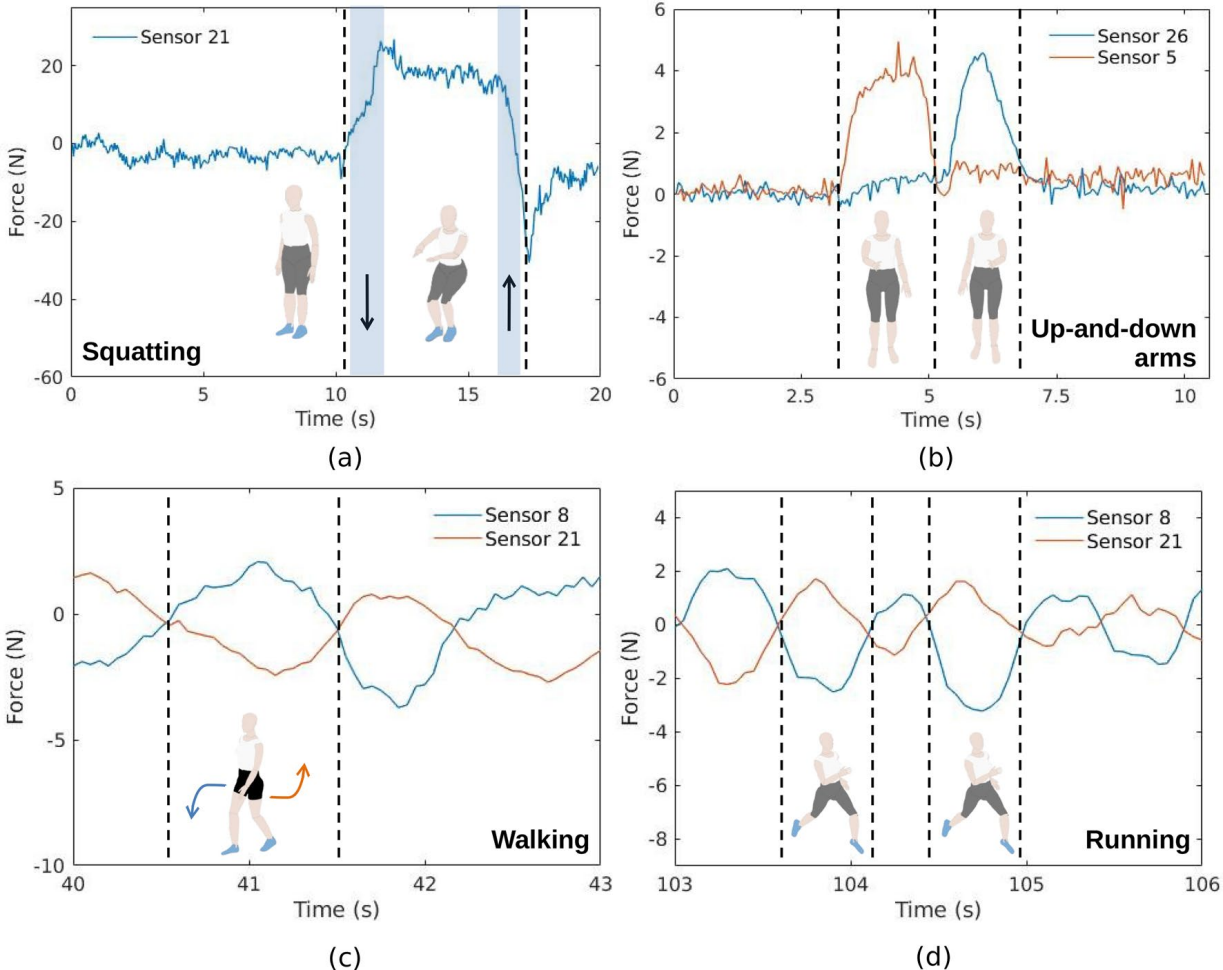
Mechanical analysis of different sensors during body moving in the different dynamic activities. (**a**) Squatting. (**b**) Up-and-down arms. (**c**) Walking. (**d**) Running.

The behavior of the sensors’ responses (especially the ones presented in Figs. 5 and 3) are related to the movement performed by the body, which leads to reaction forces in the sensors. As the sensors are characterized as a function of the applied force (see Fig. 1), it is possible to provide a mechanical analysis of the performed movement with the reaction forces on each sensor. For this reason, additional experimental results are analyzed and Fig. 6 presents the sensors’ responses (converted to force) during the four dynamic activities: squatting, up-and-down arms, walking, and running for the mechanical analysis considering the sensors with the highest activation (as previously discussed). Since the sensors are fixed in predefined regions in the garment (as shown in Fig. 1), the movements of the body during activities lead to force application on each sensor depending on the activity. For example, in the squatting activity, shown in Fig. 6a, there are activation in the lumbar and abdominal regions, leading to a force increase on the sensor 21, whereas the adjacent sensors (10, 11 and 20) also presented increase in the reaction force. In the up-and-down arms activity, presented in Fig. 6b, there is an expected variation of the reaction forces in the sensors positioned on the shoulders (sensors 5 and 26). Moreover, walking (Fig. 6c) and running (Fig. 6d) activities involve hip rotation and oscillatory movements on the trunk, where the difference between both activities is on the movement amplitude and frequency, which are converted in reaction forces in the sensors with similar behavior when compared with trunk dynamics during gait in the coronal plane^35^.

**Figure 7:**
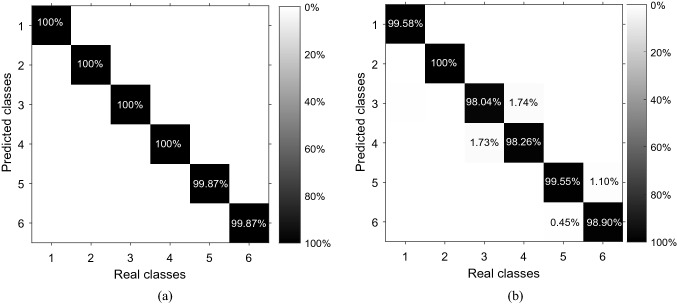
Results of the confusion matrices for volunteer 1 during activities. (**a**) Complete structure. (**b**) Optimized structure.

In order to analyze the demand of the sensors’ amount in the activities classification, the attributes are reduced and the classification performance is calculated. By applying the PCA technique to the volunteer 1 dataset, the attributes were reduced to 10 new attributes, obtained by the linear combination of the 30 sensors’ response, and presented a variance explained of 99.84%. The mean of the classification accuracy for 10 trials using the new 10 attributes was 99.02 (0.16)%, which demonstrates that a dataset linearly uncorrelated and 3 times smaller (with better processing performance) still presents a high accuracy (approximately 99%). By analyzing the eigenvectors matrix (V) used by the PCA technique, which represents the coefficients of the linear combination of the original attributes to compose this new dataset (10 attributes) and hence represents the weight of each attribute (sensor), and considering the most significant coefficients, the original attributes were arranged in descending order. By selecting the most significant 14 attributes, the accuracy is 99.71 (0.08)% in 10 trials. By selecting the most significant 12 attributes, the accuracy is 99.46 (0.17)% in 10 trials. Finally, when the most significant 10 attributes are selected the accuracy decreases to 99.05 (0.10)% in 10 trials. It means that the reduction of the smart garment to 10 sensors still provides an accuracy close to 99%, since the 10 sensors cover the body regions which move effectively in these activities for this volunteer in particular. The selected sensors were: 3, 5, 8, 11, 14, 17, 20, 23, 26 and 28. In this way, it is possible to optimize the number of smart garment sensors according to each volunteer, by analyzing their results in a predefined activity, since each person performs the movement differently. To verify the behavior of the smart garment with optimized number of sensors for volunteer 1, movement classification analyses are performed with the same conditions. Table 2 shows the accuracy, recall and precision of the complete structure and the proposed structure with smaller number of sensors in the photonic smart garment (i.e., only sensors 3, 5, 8, 11, 14, 17, 20, 23, 26 and 28, positions according with Fig. 1) for the volunteer 1. Comparing data from volunteer 1 with complete structure and data with optimized sensors in Table 2, there is a higher accuracy of the system using the 30 sensors. However, the difference between the complete structure (30 sensors) and the optimized one (10 sensors) is only 0.91%, which indicates that the optimized structure has similar performance when compared to the complete one even with fewer sensors. In order to verify the performance of the optimized structure, the additional experiments result in the confusion matrices presented in Fig. 7, where Fig. 7a represents the results with the complete structure and Fig. 7b represents the results with the optimized structure. These results show that even with a significant reduction in the number of sensors, the optimized structure still provide high accuracy for all classes, where a accuracy higher than $98\%$ was obtained. Thus, it is possible to assume that the proposed optimization of the number of sensors does not impose major influence on the photonic smart garment repeatability and overall performance and can be performed for the on-body optimization of the structure for each user or group of users.

**Table 2:** Comparison between the classification results for volunteer 1 with complete structure and optimized structure.

Volunteer	Complete structure	Optimized structure
Accuracy (%)	99.96 (0.04)	99.05 (0.10)
**Recall (%)**		
$c_1$	100 (0.00)	99.46 (0.21)
$c_2$	100 (0.00)	100 (0.00)
$c_3$	100 (0.00)	97.92 (0.51)
$c_4$	100 (0.00)	98.43 (0.30)
$c_5$	99.87 (0.00)	99.38 (0.19)
$c_6$	99.87 (0.00)	99.08 (0.24)
**Precision (%)**		
$c_1$	100 (0.00)	99.64 (0.22)
$c_2$	100 (0.00)	100 (0.00)
$c_3$	100 (0.00)	97.85 (0.26)
$c_4$	100 (0.00)	98.32 (0.44)
$c_5$	99.87 (0.00)	99.09 (0.24)
$c_6$	99.87 (0.00)	99.37 (0.20)

**Figure 8:**
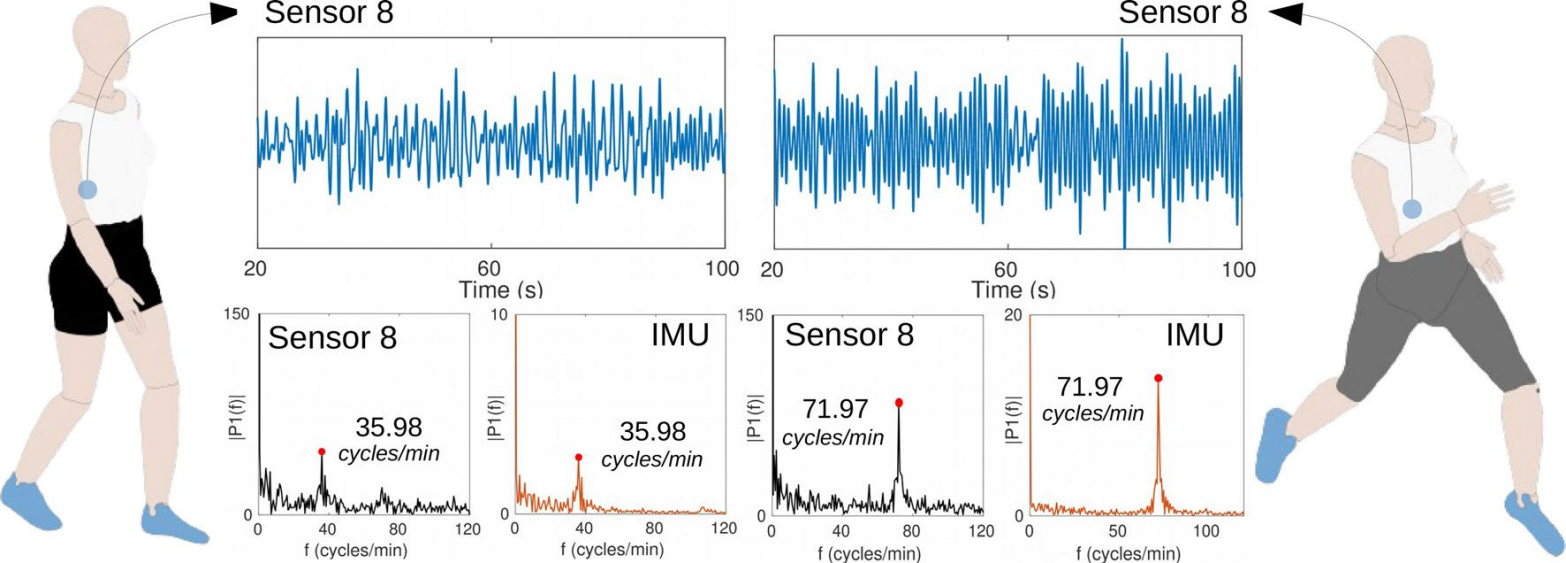
Results of walking and running tests of the volunteer 1 for cadence estimation: temporal response and FFT of the IMU data (yaw) and the response of sensor 8.

In order to estimate the **cadence**, the sensors’ responses during the walking and running activities were analyzed. An inertial measurement unit (IMU) was incorporated in the upper back of the garment as a reference system for the comparison with the proposed smart garment, and the yaw data obtained from the IMU were compared with the photonic smart garment (sensor 8, since this sensor is located on the lateral of the body and presents a great variation in the response during walking and running). By applying the FFT to the temporal response of the yaw data and the one of sensor 8 (positioned on the lower right side of the body), both results presented frequency peaks of 35.98 cycles/min and 71.97 cycles/min, as shown in Fig. 8. These frequencies correspond to cycles of stride per minute, which leads to a double value of cycles of steps per minute, since a stride consists of two steps. Thus, by analyzing the FFT results, the estimated cadence is 71.96 steps/min (walking) and 143.94 steps/min (running), presenting no error between the results of photonic smart garment sensors and the IMU.

**Figure 9:**
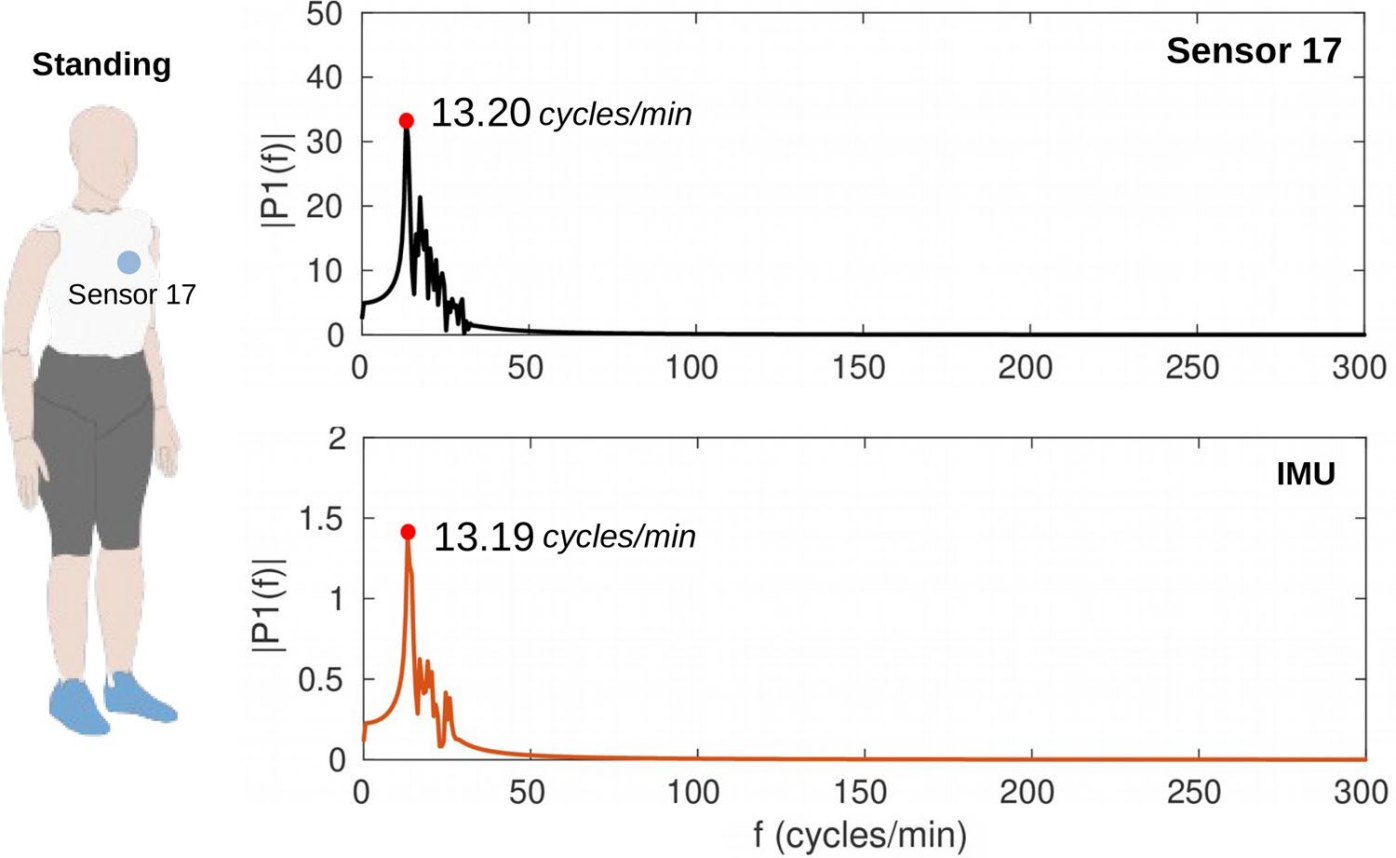
FFT of the IMU data (pitch) and the response of sensor 17 during standing activity of the volunteer 1 for breathing rate estimation.

In order to estimate the **breathing rate**, the sensors’ responses during the standing test were analyzed. The volunteer was in upright position and performed no movement to reduce the influence of artifacts. A 0.3–0.7 Hz bandpass filter was applied to attenuate external noises. The pitch data obtained by the IMU were compared with the smart garment (sensor 17, since this sensor is positioned on the middle of the body and presents a high variation during the breathing task). By applying the FFT on the temporal response of sensor 17 and pitch data, results presented peaks of 13.20 and 13.19 cycles/min, respectively, which lead to the estimated breathing rates of 26.40 and 26.38 cycles in 2 minutes, as presented in Fig. 9. The estimated breathing rates from IMU and photonic smart garment data present high correlation (0.08% relative error). Table 3 shows the estimated cadence and breathing rate for all volunteers. The errors between the parameters estimated by the photonic smart garment sensors and by the IMU occur due to the cross sensitivity of the POF sensors, whereas the IMU is a 3D sensor and the parameters extraction involves data from only one dimension, which reduces the interference of the data from another axes, decreasing the noise in the FFT analysis.

**Table 3:** Estimated parameters from IMU (reference) and Photonic Smart Garment sensors: errors between the measurements obtained from the two systems.

Volunteer	IMU			Smart Garment			Errors		
	Cadence (steps/min)		BR (cycles/min)	Cadence (steps/min)		BR (cycles/min)	Cadence (%)		BR (%)
	Walking	Running	Standing	Walking	Running	Standing	Walking	Running	Standing
1	71.96	143.94	13.19	71.96	143.94	13.20	0	0	0.08
2	79.14	155.92	13.79	77.38	156.54	13.9	2.22	0.40	0.80
3	68.36	125.94	13.79	68.60	126.64	14.04	0.35	0.56	1.81
4	73.16	146.34	14.39	72.12	145.98	14.6	1.42	0.27	1.46

**Figure 10:**
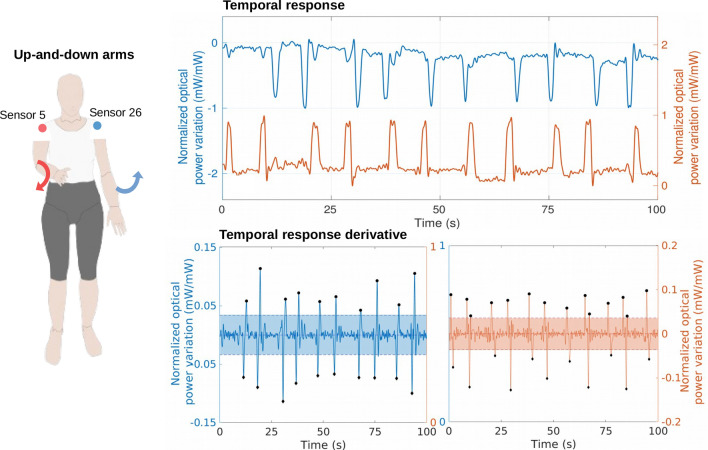
Results of the up-and-down arms test of the volunteer 1: temporal response of sensors 5 (right arm) and 26 (left arm) and identification of the shoulder flexion and extension by outliers detection using the temporal response derivative.

The assessment of the **arms movement** was based on the temporal analysis of sensors 5 (right arm) and 26 (left arm) since these sensors are located close to the shoulders and they present a high optical power variation during the up-and-down arms. The responses of both sensors were normalized and can be observed in Fig. 10. The first curves represent the flexion and extension of each arm. In order to identify the moment of the shoulder flexion and extension, an outlier detection algorithm was employed on the derivative of the sensors’ temporal responses (5 and 26); results of the outlier detection are presented in the bottom graphics of Fig. 10. The shaded areas limited by the dotted lines represent the natural movement range based on the *z-score* calculation ($mean \pm 3 \cdot standard~deviation$)^36^, whereas the markers located outside the areas represent the moment of flexion and extension of the shoulders (outliers).

## Discussions

In this paper, a photonic smart garment based on 30 multiplexed POF sensors for activity identification and movement-related parameters extraction was proposed. The system includes a quasi-distributed sensor system, i.e. a system with several measurements points along the POFs, and wireless communication, which leads to a fully portable system optimized for IoT applications, including remote healthcare monitoring. In contrast with other quasi-distributed optical fiber sensors, the proposed approach has low cost, a compact signal acquisition system and a simple sensor fabrication process, differently from FBGs which need specialized equipment for the sensor fabrication, in addition to high cost and bulky interrogators for the signal acquisition.

A human activity recognition protocol is proposed to evaluate the ability of identifying the human motor activity using the photonic smart garment. Six daily living tasks were performed by 4 volunteers for 2 minutes. The kNN classifier was used due to its simple implementation and significant classification performance, in addition to the fact that the data are simple and organized, which did not lead to the need for more advanced techniques such as neural networks. The kNN classifier was based on the dataset randomly permuted and divided into training (70%) and testing (30%). This classification approach was repeated and the results of 10 trials presented a classification accuracy of 94.00 (0.14)%, across volunteers. The demand of the number of sensors was evaluated by using the dimensionality reduction technique PCA in volunteer 1 dataset. New attributes resulting from the linear combination of the original dataset were obtained and results showed an average accuracy across subjects of 99.02 (0.16)% using only 10 new attributes. From these 10 attributes, the weight of the original features were evaluated and the attributes with lowest weights were rejected. Thus, the most significant 10 original attributes (covering all the body regions) presented an accuracy of 98.14 (0.31)%, thus maintaining high accuracy even with a reduced number of sensors, which facilitates the fabrication process and improves the real-time performance in a remote healthcare monitoring.

Finally, movement-related parameters were extracted and validated during the human activity recognition protocol. The arms movement was analyzed during the up-and-down arms activity and the flexion and extension of left and right arms were identified by using an outlier technique detection of the sensors temporal derivative. Cadence and breathing rate parameters were validated by using an IMU positioned on the upper back region. The cadence was evaluated during the walking and running activities and presented errors of 1.00 (1.01)% and 0.31 (0.24)%, respectively, when compared with the cadence estimated by the IMU data. The breathing rate parameter was evaluated during the standing activity in which no movement is involved and the photonic smart garment sensors presented an error of 1.04 (0.76)% from the result of the IMU. The low mean and standard deviation of the errors indicate good accuracy and repeatability of the system to measure the different parameters. In addition, the proposed system proved to be a feasible option to extract different types of biomedical parameters using an instrumented lightweight clothing which can be employed in the daily activities without disturbing the user’s movement. Therefore, the proposed approach is an optimized option for remote healthcare applications to identify activities and extract different parameters using low cost and compact components integrated in an usual clothing. Future works include the application of the whole system in a real-time healthcare monitoring to be used in a clinical assessment.

## Methods

### Smart garment development

The photonic smart garment comprises of 30 multiplexed intensity variation-based POF sensors incorporated in a vest. In addition, an IMU MTi-3 (Xsens Technologies B.V., NL) is positioned on the upper region of the smart garment back to estimate the trunk angles. The POF is made of polymethyl methacrylate, PMMA (HFBR-EUS100Z, Broadcom Limited) with a core diameter of 980 μm, a cladding of fluorinated polymer with 20 μm thickness and a polyethylene coating, resulting in a total diameter of 2.2 mm. The 30 sensors are divided in 4 POFs and the optical power is acquired by four photodetectors IF-D92 (Industrial Fiber Optics, Tempe, AZ, USA) positioned at one end of each fiber. To increase the transmitted optical power, four 3D facets with aluminum foil are positioned at the other end of each fiber, in which the light reflects in the aluminum foil towards the photodetector direction.

**Figure 11:**
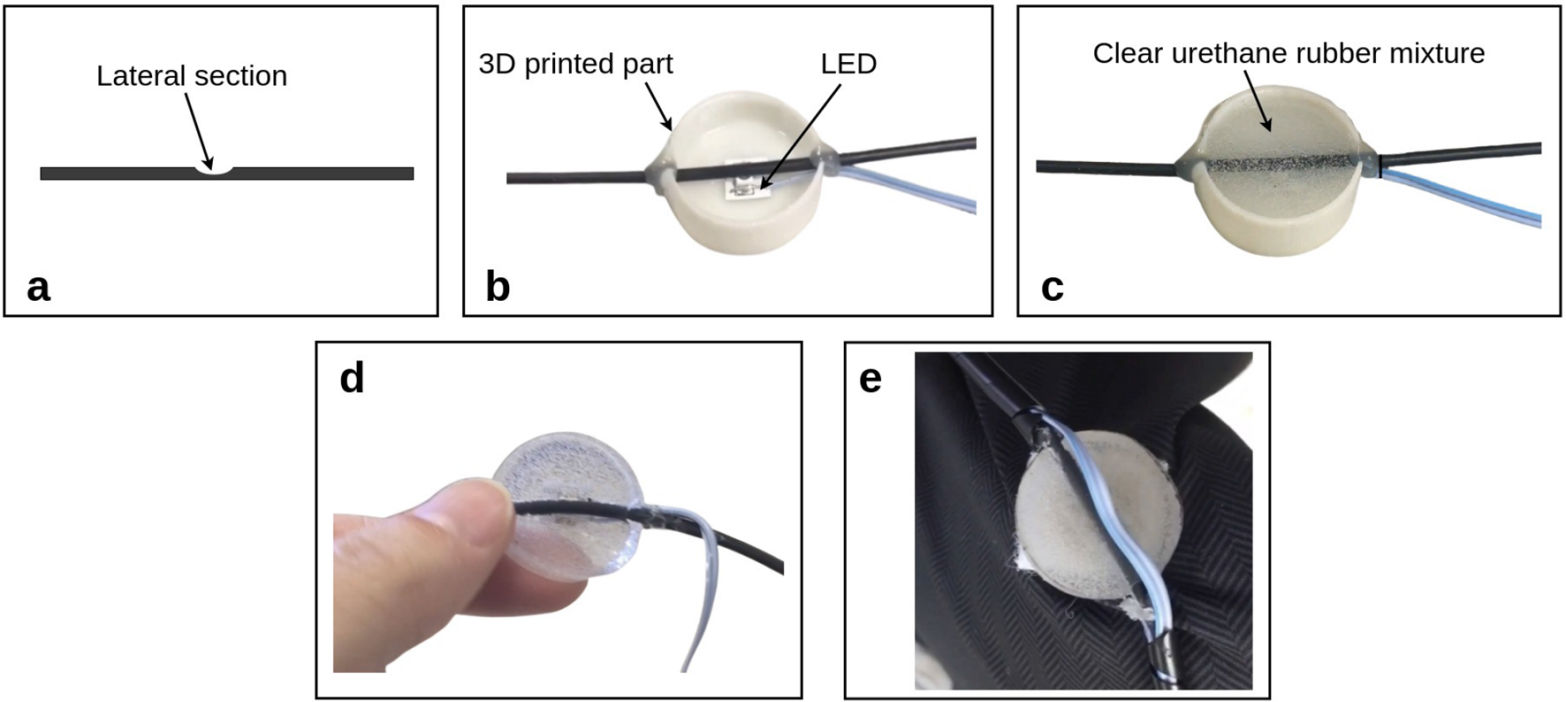
Sensors fabrication process. (**a**) Removal of part of the fiber material creating a lateral section. (**b**) LED coupling to the fiber lateral section in a 3D printed part. (**c**) Sensor encapsulation using clear urethane rubber mixture. (**d**) Encapsulated sensor. (**e**) Sensor incorporated in the garment.

As shown in Fig. 11, the sensors fabrication process is divided into 3 stages: (i) lateral section creation by removing part of the fiber material (see Fig. 11a), (ii) LED coupling to the sensitive zone (see Fig. 11b) and (iii) sensor encapsulation using clear urethane rubber mixture in a 3D printed part (see Fig. 11c). The fiber lateral section is created through a curved razor blade and afterwards, the fiber is attached to the 3D printed part (diameter = 30mm / height = 7mm) with the lateral section pointed towards the LED, which is already attached to the 3D part base. With the fiber lateral section coupled to the LED, the clear urethane rubber mixture is spilled into the part and reserved for 24h at room temperature to be cured. Figure 11d presents the encapsulated sensor when the clear urethane rubber is cured and Fig. 11e shows the sensor incorporated in the textile. The connection between the optical fiber is achieved by the clear urethane rubber as shown in Fig. 11d, where the layer between the optical fiber and LED is filled with the urethane rubber. The clear rubber has an attenuation of around 0.7*dB*/*cm*, which leads to a light loss of around 0.35*dB* in the 5mm distance between the LED and optical fiber. The stages i, ii and iii can result in sensor-to-sensor differences. The length and depth of the lateral section are controlled using a manufactured mold for the razor blade used in the material removal. Moreover, the 3D-printed part has the supports for the optical fiber and the LED to control the coupling distance between the LED and the optical fiber to minimize the errors in the lateral section parameters and side-coupling. However, there are minor deviations in the lateral section parameters, the coupling distance and the clear urethane rubber mixture that affect the sensor sensitivity. In order to mitigate the effect of the different sensors sensitivities in the activity analysis, all sensors are characterized in terms of the applied force and each sensor is normalized by its own sensitivity. The temperature variations can influence the sensors responses as previously discussed in the literature^30^. However, the temperature sensitivity (0.0014 a.u./$^\circ \hbox {C}$) of the proposed sensors is smaller than the mean force sensitivity (around 0.0089 a.u./N), which leads to lower influence on the temperature response. In addition, the tests are performed in room temperature conditions, where the temperature variation dynamics is order of magnitude smaller than the dynamic movements, which leads to the possibility of mitigating the temperature influence in the sensors responses using filtering techniques^37^. Moreover, it is also possible to use some of the sensors in the smart garment (e.g. the ones with smaller optical power variation in the activities protocol) for temperature compensation of the sensors responses using the temperature sensitivity of all sensors through the direct difference method^38^. However, the sensors are in contact with the human body, which presents almost constant temperature. It leads to a low influence of temperature on the sensors.

### Human activity recognition protocol

The human activity recognition protocol consists of 4 healthy volunteers wearing the smart garment and performing six different daily activities: standing, sitting, squatting, up-and-down arms, walking and running. This protocol aims to monitor common daily movements and use the smart garment to identify each activity in addition to extract different movement-related parameters during the tests.

Standing represents the activity in which the volunteer is in upright position, whereas the sitting activity involves the volunteer sitting on a chair. The squatting activity is divided into 5 stages (states and transitions): (i) upright position (state), (ii) squatting down (transition between upright to squatting), (iii) squatting (state), (iv) squatting up (transition between squatting to upright) and (v) upright position (state), and these stages are cyclically repeated. The up-and-down arms activity is divided into 4 stages (states and transitions): (i) moving up the right arm (shoulder flexion of 90$^\circ$), (ii) moving down the right arm (shoulder extension of 90$^\circ$, back to neutral position), (iii) moving up the left arm (shoulder flexion of 90$^\circ$) and (iv) moving down the left arm (shoulder extension of 90$^\circ$, back to neutral position), and they are cyclically repeated. Finally, the walking and running activities were performed on a treadmill to control the velocities and achieve a movement pattern. The walking speed is 0.5 m/s and the running speed is 2.0 m/s. All activities were performed for 2 minutes. The tests were performed in accordance with the guidelines of the national health council with the protocols approved by Research Ethics Committee through the National Commission in Research Ethics-CONEP-(Certificate of Presentation for Ethical Appreciation-CAAE: 41368820.3.0000.5542) in March, 2020. In addition, written informed consent was obtained from each subject prior to data collection.

### Data processing and Machine Learning training model

The multiplexing technique proposed by^30^ consists of a sequential activation of the LEDs and the optical power acquisition in a short time interval. After the activation and optical power acquisition of the last LED, the optical power of all sensors is transmitted to a microcontroller and this process is cyclically repeated. The sensors’ signal results in a matrix with 30 columns (corresponding to the 30 sensors) and n rows (corresponding to the n samples).

For the activity classification, the kNN classifier was employed. The kNN classifier is a popular method with simple implementation and significant classification performance^34^. Furthermore, the kNN is a supervised method of the machine learning field which does not assume a linear class boundary, since the kNN method determines the class based on the k-nearest neighbor training points. For this reason, it has the advantage of producing classification fits that adapt to any boundary^39^. The kNN input data comprise 30 attributes (response of the 30 sensors), six classes (activities) and each sample is labeled according to the respective activity. The classes are divided into: standing ($c_1$), sitting ($c_2$), squatting ($c_3$), up-and-down arms ($c_4$), walking ($c_5$) and running ($c_6$). Thus, all data are randomly permuted and divided into training (70%) and testing (30%). The classification processing is repeated for 10 times. Accuracy, recall, precision and confusion matrix are used as model evaluation metrics, comparing the real labels with the labels estimated by the kNN model.

In order to reduce the dimensionality of the model attributes, the PCA technique is employed. PCA is not an attribute selection technique, but a technique from the field of linear algebra which converts an attribute set to a new dataset linearly uncorrelated, obtained from the linear combination of the original attributes, so-called principal components. As the principal components have a sample-like pattern with a weight for each attribute, we can use the weights to visualize the influence of each attribute on the dataset^40^.

Considering the matrix $X_{nxD}$, in which the rows represent the samples and the columns represent the attributes, each sample is normalized by subtracting the attribute mean ($\mu _j$) and dividing by the attribute standard deviation ($\sigma _j$), as shown in Eq. (1).

1 $$\begin{aligned} {\hat{x}}_{i,j} = \frac{ x_{i,j} - \mu _{j} }{ \sigma _{j} }, \quad j = 1,2,\ldots ,d \quad i=1,\ldots ,N \end{aligned}$$

With the new matrix X (with mean 0), the correlation matrix C is calculated by using the Eq. (2). From this matrix, the eigenvalues ($\lambda$) and eigenvectors (V) are obtained.

2 $$\begin{aligned} C = \frac{ 1 }{ (N-1) } {\hat{X}}^T {\hat{X}} \end{aligned}$$

For the dimensionality reduction, the k eigenvectors associated to the largest eigenvalues are selected to compose the new dataset ($\Omega _x$), calculated by the Eq. (3). The k eigenvectors are defined by analyzing the variance explained by each principal component, in which the variance might be higher than 99%, and the number of attributes is reduced.

3 $$\begin{aligned} \Omega _{X}^{T} = X \cdot V_{Nxk} \end{aligned}$$

Also, by analyzing the eigenvectors matrix (V), it is possible to obtain the negligible values associated to the original attributes (sensors) in the calculation of the new attributes^40^, and the possibility of reducing the number of sensors while maintaining the algorithm classification performance, resulting in an optimized photonic sensor system.

### Human movement-related parameters extraction

In addition to the ability of classifying the human activities, several movement-related parameters can be extracted by analyzing the sensors’ responses of the smart garment. During the walking and running activities, the responses of the smart garment sensors and the IMU data (reference) were analyzed. The fast Fourier transform (FFT) of the sensors’ temporal response is applied for the cadence estimation. In the up-and-down arms activity, by analyzing the arms movement it is possible to monitor the flexion and extension of each shoulder and evaluate the similarity or not between both sides. An outlier detection algorithm based on *z-score* calculation^36^ is applied on the derivative of the sensors’ temporal responses and the identified outliers represent the moment of flexion and extension of each shoulder. During the standing activity, which does not involve movement and the breathing is uniform, the breathing rate was estimated using the fast Fourier transform (FFT) of the sensors’ temporal response of IMU data (reference) and the photonic smart garment sensors.

## Supplementary Information

Supplementary Information.
