# Supplementary material for: AI-enabled photonic smart garment for movement analysis
Source: Sci Rep. 2022 Mar 8;12:4067. doi: 10.1038/s41598-022-08048-9 (PMC8904460; doi:10.1038/s41598-022-08048-9)
Supplement: Supplementary file 1 — Supplementary Information. [file 41598_2022_8048_MOESM1_ESM.docx]

**Supplementary Information**

**AI-enabled Photonic Smart Garment for Movement Analysis**

Leticia Avellar^1,*^, Carlos Stefano Filho^2^, Gabriel Delgado^3^, Anselmo Frizera^1^, EduardoRocon^3^, and Arnaldo Leal-Junior^1^

^1^Graduate Program in Electrical Engineering, Federal University of Espírito Santo (UFES), Fernando Ferrari Avenue, Vitória 29075-910, Brazil.

^2^Neurophysics Group, “Gleb Wataghin” Institute of Physics, University of Campinas, Brazil.

^3^Centro de Automática y Robótica, Ctra. Campo Real, 28500 Arganda del Rey, Madrid, Spain.

[**^*^leticia.avellar@aluno.ufes.br**](mailto:*leticia.avellar@aluno.ufes.br)

Table S1 presents the data (samples) that resulted in the confusion matrix in Figure 4. The data consist of the number of samples that correspond to each class, in which the columns represent real classes and the rows represent predicted classes. The matrix diagonal consists of correctly classified samples.

**Table S1.** Data that resulted in the confusion matrix in Figure 4.

|  |  | Real classes | | | | | |
| --- | --- | --- | --- | --- | --- | --- | --- |
|  |  | c1 | c2 | c3 | c4 | c5 | c6 |
| Predicted classes | c1 | 32515 | 9 | 419 | 325 | 98 | 33 |
|  | c2 | 17 | 33308 | 76 | 123 | 20 | 90 |
|  | c3 | 62 | 9 | 30573 | 1010 | 197 | 540 |
|  | c4 | 151 | 20 | 1054 | 32379 | 16 | 281 |
|  | c5 | 2 | 5 | 343 | 43 | 25799 | 3724 |
|  | c6 | 1 | 11 | 383 | 66 | 2229 | 23399 |
